# Supplementary material for: Insights into energy balance dysregulation from a mouse model of methylmalonic aciduria
Source: Hum Mol Genet. 2023 Jun 27;32(17):2717–34. doi: 10.1093/hmg/ddad100 (PMC10460489; doi:10.1093/hmg/ddad100)
Supplement: SI_revised_v3_ddad100 [file si_revised_v3_ddad100.docx]

**Supplementary Information**

Insights into energy balance dysregulation from a mouse model of methylmalonic aciduria

Marie Lucienne^1,2,3 a^, Raffaele Gerlini^4 a^, Birgit Rathkolb^4,5,6^, Julia Calzada-Wack^4^, Patrick Forny^1^, Stephan Wueest^7^, Andres Kaech^8^, Florian Traversi^1^, Merima Forny^1^, Céline Bürer^1^, Antonio Aguilar-Pimentel^4^, Martin Irmler^4^, Johannes Beckers^4,6^, Sven Sauer^9^, Stefan Kölker^9^, Joseph P. Dewulf^10,11,12^, Guido T. Bommer^10,11^, Daniel Hoces^13^, Valerie Gailus-Durner^4^, Helmut Fuchs^4^, Jan Rozman^4,6^, D Sean Froese^1,2 b^, Matthias R. Baumgartner^1,2,3 b*^, Martin Hrabě de Angelis^4,6,14 b^

1. Division of Metabolism and Children’s Research Center, University Children’s Hospital Zurich, University of Zurich, Zurich, Switzerland
2. radiz – Rare Disease Initiative Zurich, Clinical Research Priority Program for Rare Diseases, University of Zurich, Zurich, Switzerland
3. Zurich Center for Integrative Human Physiology, University of Zurich, Zurich, Switzerland
4. Institute of Experimental Genetics and German Mouse Clinic, Helmholtz Zentrum München, German Research Center for Environmental Health, Neuherberg, Germany
5. Institute of Molecular Animal Breeding and Biotechnology, Gene Center, Ludwig-Maximilians-University München, Munich, Germany
6. German Center for Diabetes Research (DZD), Neuherberg, Germany
7. Division of Pediatric Endocrinology and Diabetology and Children’s Research Center, University Children's Hospital, University of Zurich, Zurich, Switzerland
8. Center for microscopy and image analysis, University of Zurich, Zurich, Switzerland
9. Division of Pediatric Neurology and Metabolic Medicine, Center for Pediatric and Adolescent Medicine, University Hospital, Heidelberg, Germany
10. Department of Biochemistry, de Duve Institute, UCLouvain, Brussels, Belgium
11. Walloon Excellence in Life Sciences and Biotechnology (WELBIO), Brussels, Belgium
12. Department of Laboratory Medicine, Cliniques universitaires Saint-Luc, UCLouvain, Brussels, Belgium
13. Institute of Food, Nutrition and Health, D-HEST, ETH Zurich, Zurich, Switzerland
14. Chair of Experimental Genetics, School of Life Science Weihenstephan, Technische Universität München, Freising, Germany

^a.^ These authors contributed equally

^b.^ These authors contributed equally

^*^ To whom correspondence should be addressed:

M.R.B. - Division of Metabolism, University Children’s Hospital Zurich, 8032 Zurich, Switzerland.

Tel: 41-44-266-7722; Fax:41-44-266-71-67; E-mail:matthias.baumgartner@kispi.uzh.ch.


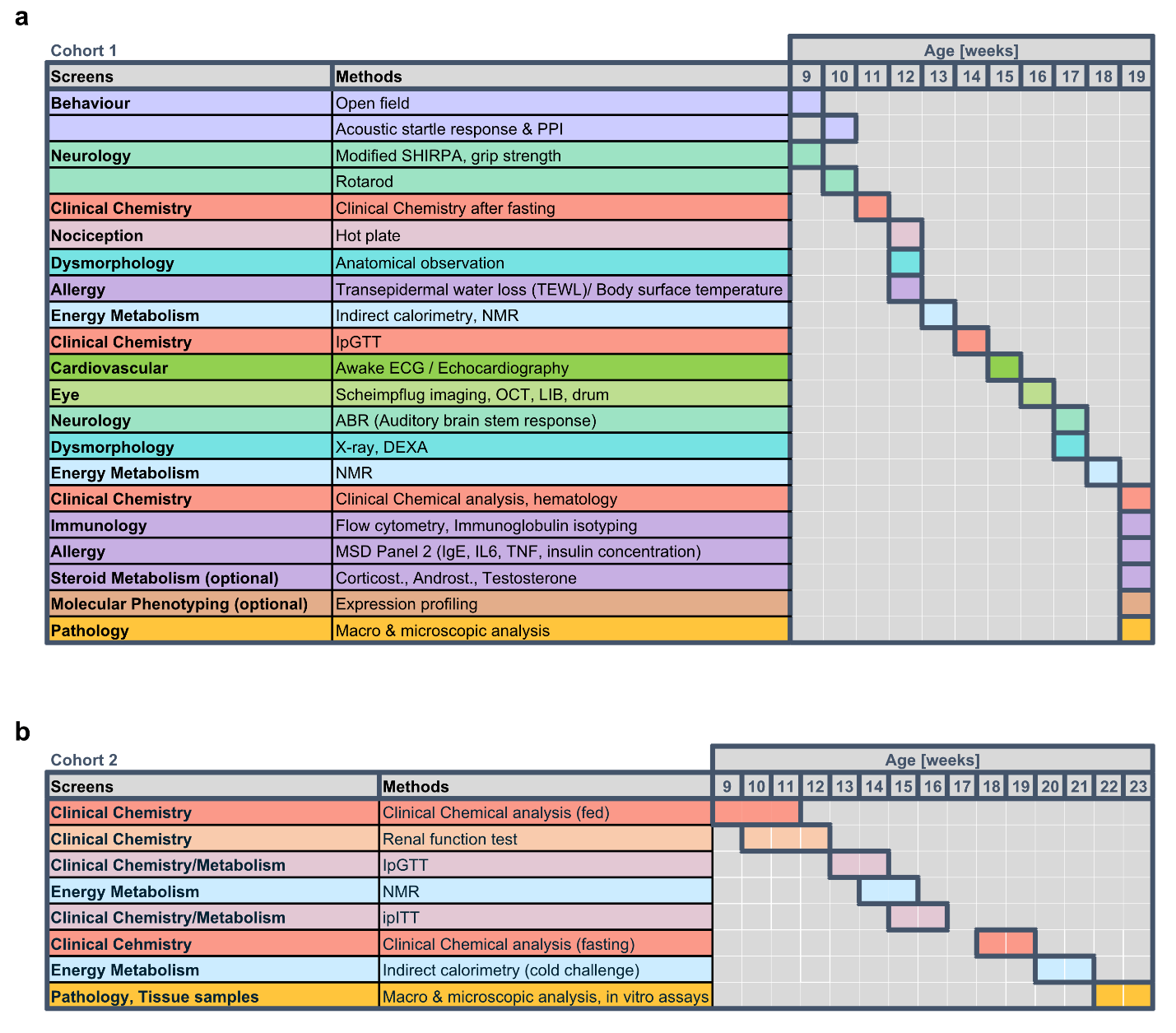


**Supplementary Figure 1. Experimental workflow of the 2 mice cohorts. a** Mice in the first cohort were examined in a an experimental workflow modified after Gailus-Durner 2009, Lucienne 2020). **b** Mice in the second cohort were examined in the depicted bespoke experimental workflow.


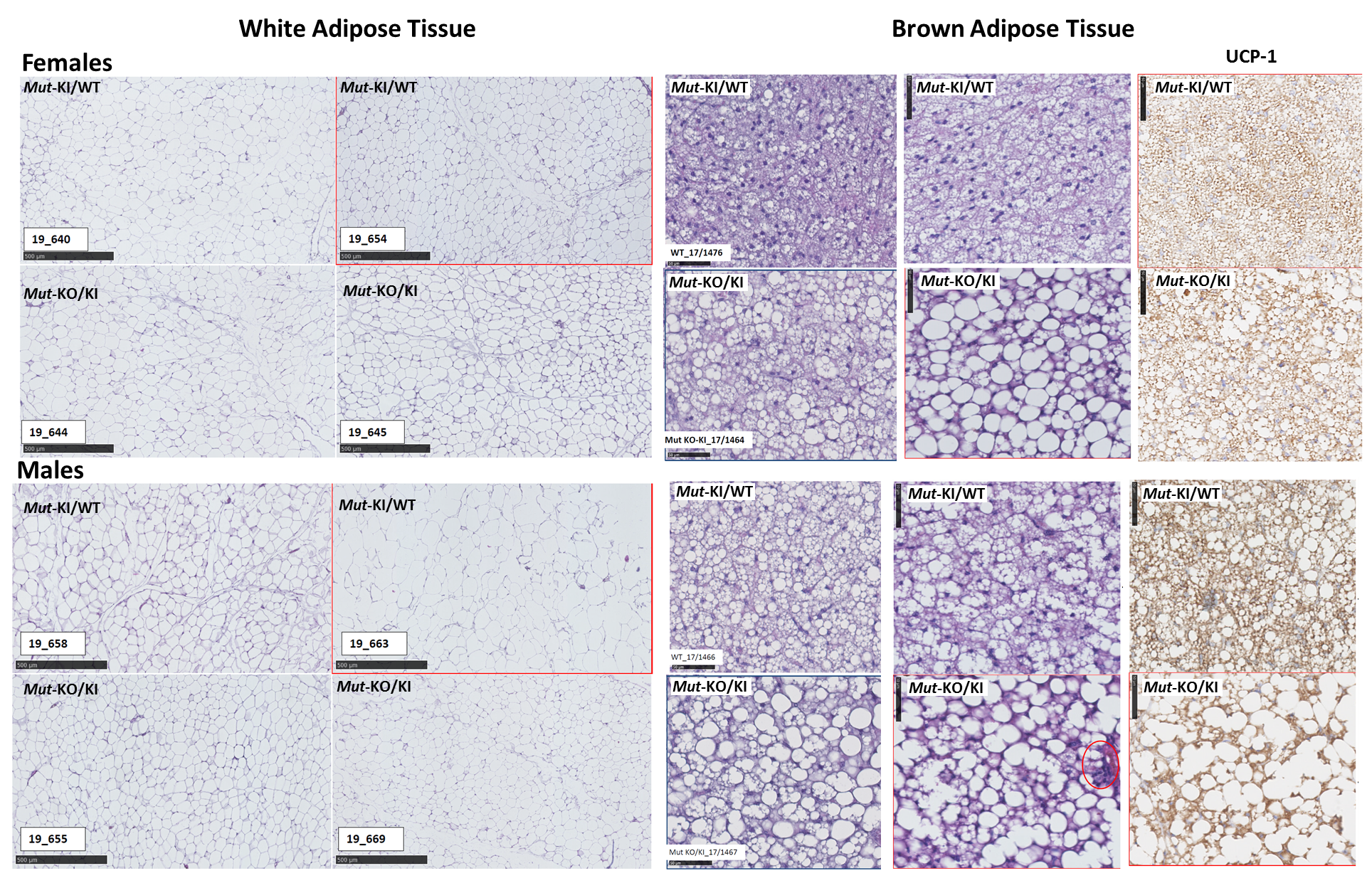


**Supplementary Figure 2. H&E staining and UCP-1 detection of white adipose tissue and brown adipose tissue.** Left side panels for both white adipose tissue and brown adipose tissue are also been displayed in Figure 2a and b.


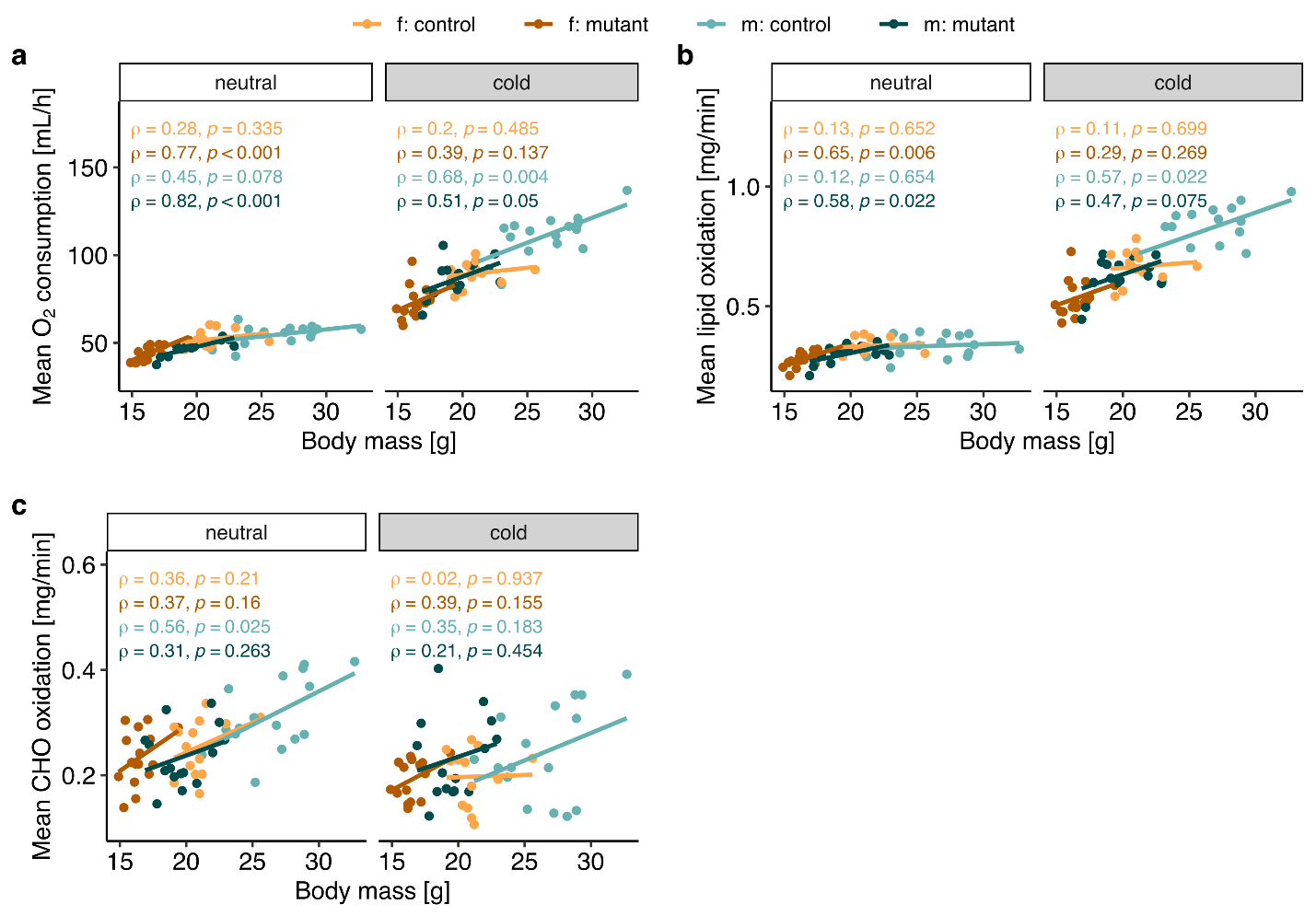


**Supplementary Figure 3. Linear models of indirect calorimetry before and during cold challenge.** **a** Oxygen consumption, **b** lipid oxidation, and **c** carbohydrate (CHO) oxidation of each mouse from Figure 3 separated according to neutral or cold environmental condition.


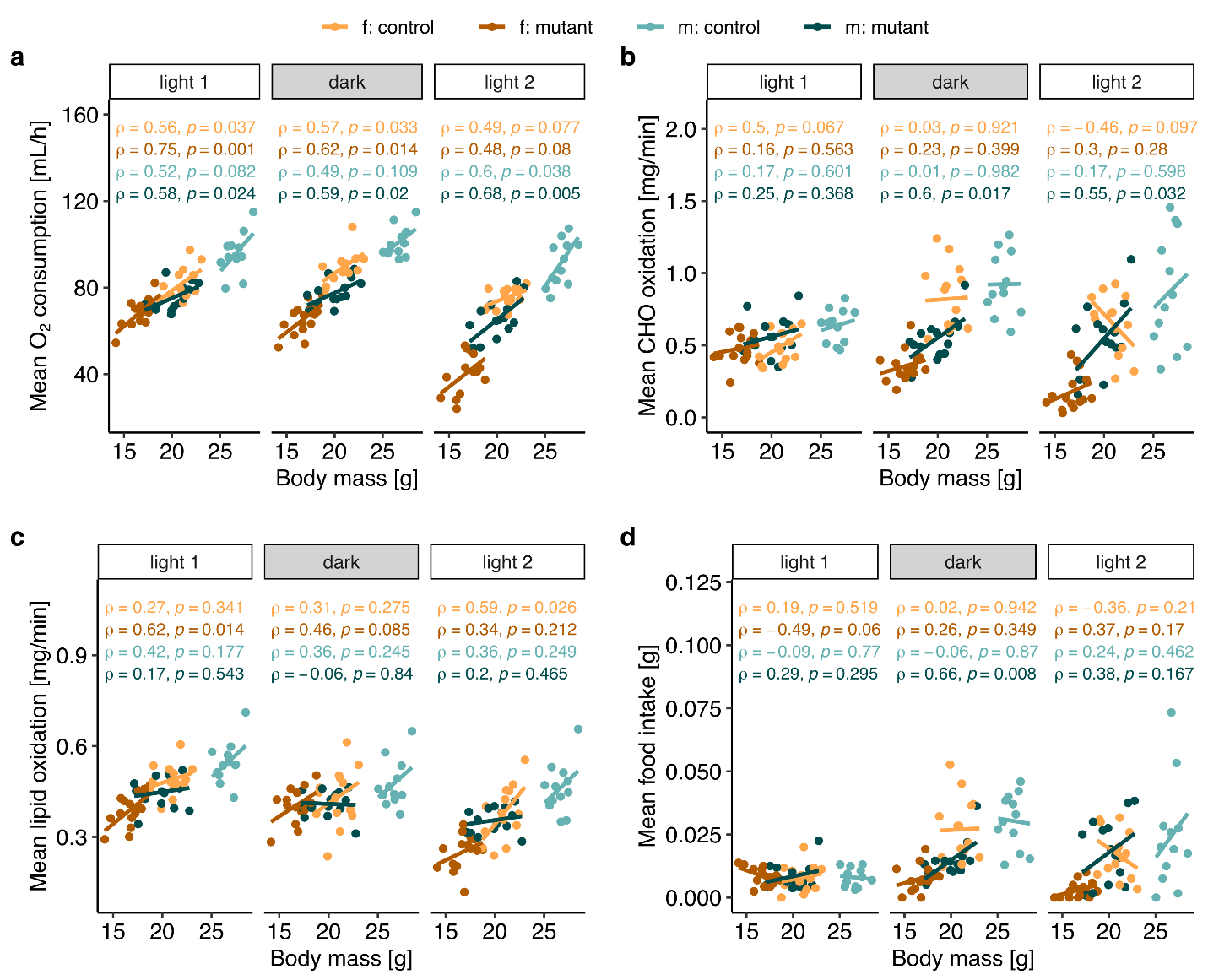


**Supplementary Figure 4. Linear models of indirect calorimetry in standard conditions.** **a** Oxygen consumption, **b** lipid oxidation, **c** carbohydrate (CHO) oxidation. **d** Food intake of each mouse from Figure 4, averaged across all time points according to light and dark phases. Therefore, each point represents average food intake per 20 minutes.


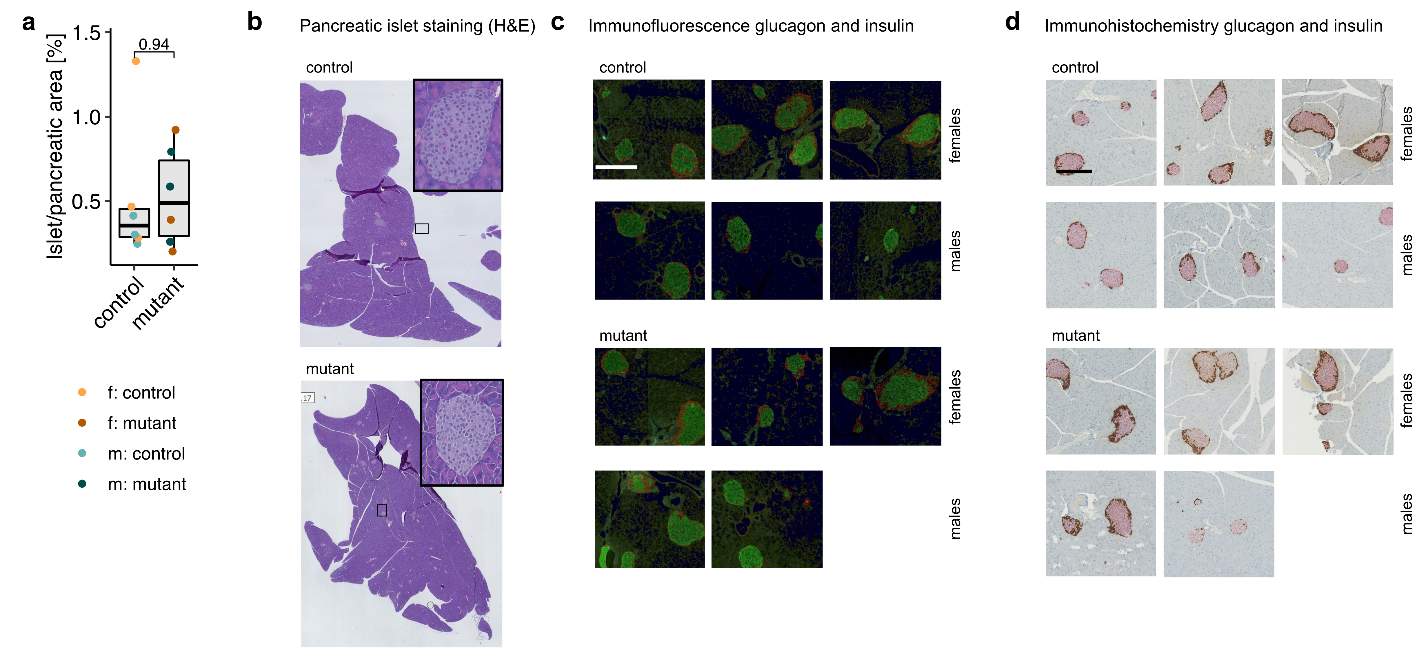


**Supplementary Figure 5. a** Ratio of islet area compared to pancreatic area determined from n=3 *Mmut*-ki/wt females, n=3 *Mmut*-ko/ki females, n=2 *Mmut*-ki/wt males, n=2 *Mmut*-ko/ki males. p-value determined by Wilcoxon rank test. **b** Representative H&E of pancreas including islet (inset) of a control and mutant mouse. **c-d** Representative islet immunohistochemistry of glucagon and insulin detected by **c** immunofluorescence (insulin: green, glucagon: red) and **d** chromogenic labelling (insulin: dark red, glucagon: light red). Each panel represents a different mouse. Scale bar represents 250 µm. (f. females, m. males, mutant: *Mmut-ko/ki*, control: *Mmut*-ki/wt)


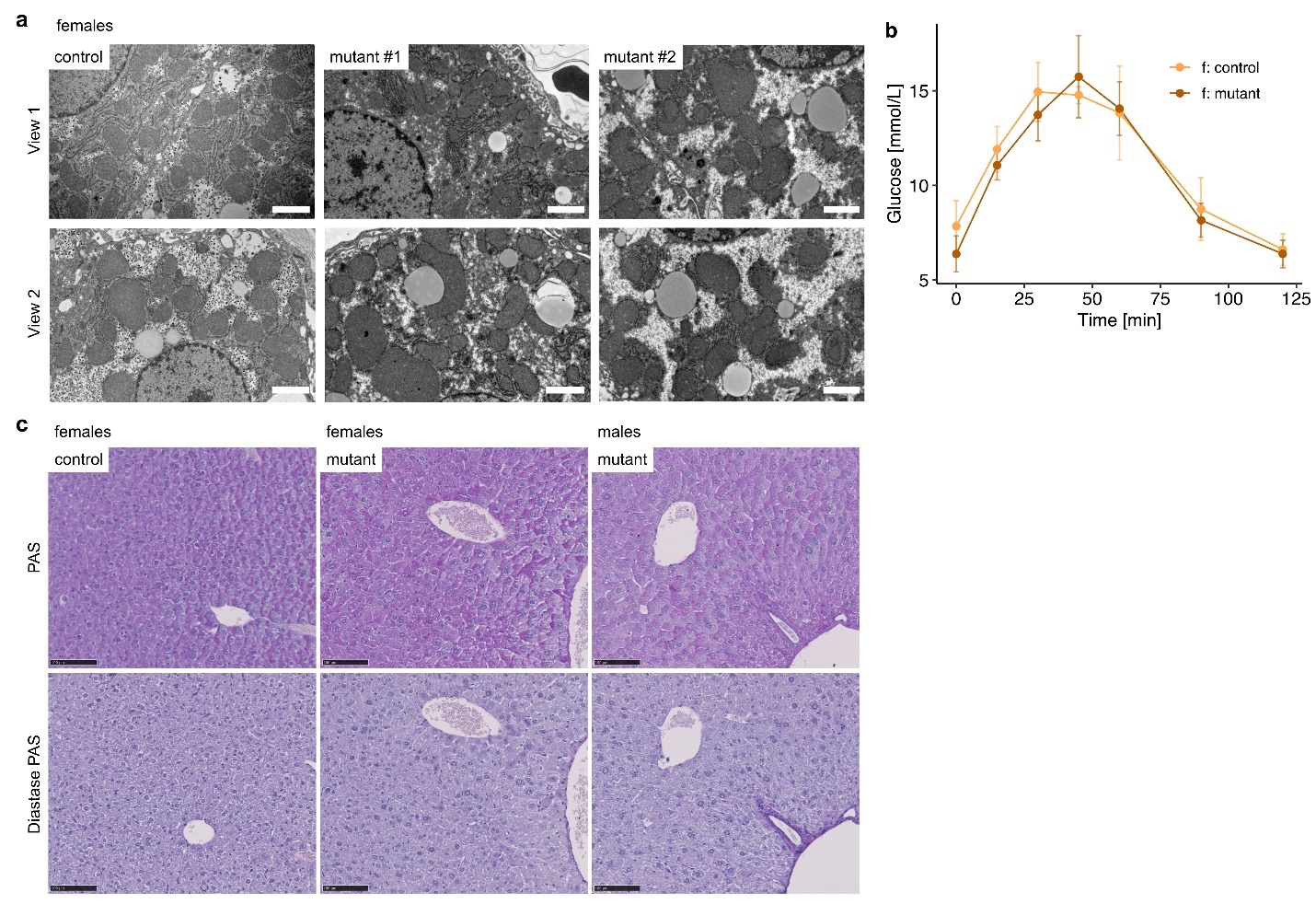


**Supplementary Figure 6. a** Transmission electron microscope images of liver samples from two distinct *Mmut*-ko/ki mice and a *Mmut*-ki/wt mouse, each 7 months of age. Scale bar represents 2 µm. **b** Glucose excursion after intraperitoneal injection of pyruvate. n=7 *Mmut*-ko/ki females and n=7 *Mmut*-ki/wt females mice, 5.5 months of age. Mean +/- SD is shown. **c** Upper panel: serial liver sections stained with PAS (periodic acid-Schiff) to detect polysaccharides such as glycogen. Magenta color represents PAS positive. Lower panel: PAS staining of the same sections following digestion with diastase (which must be PAS negative). Scale bar represents 100 µm.

**
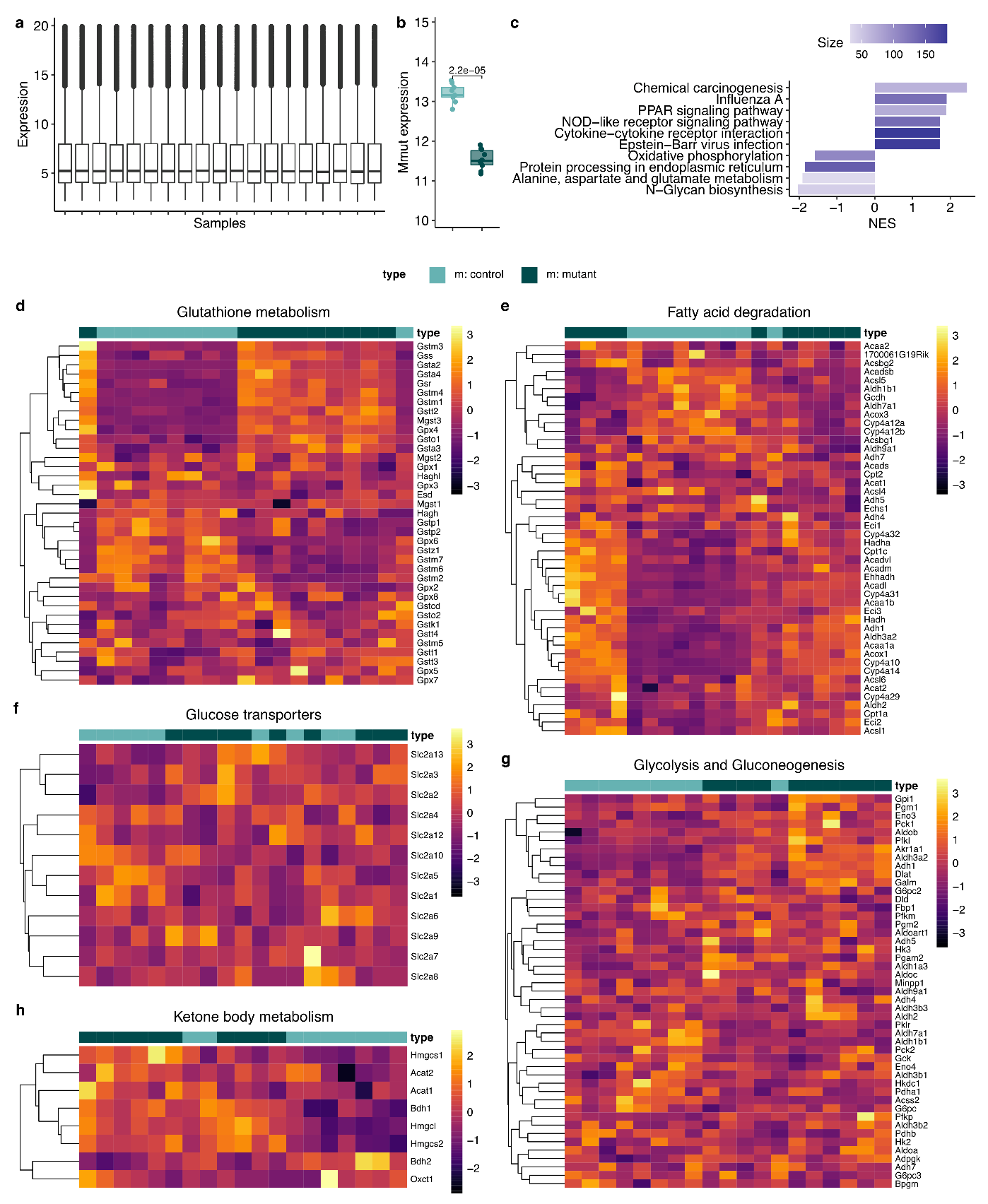
Supplementary Figure 7.** **a** Total microarray signal for each sample. **b** Expression of *Mmut* detected by microarray; p value calculated by Wilcoxon test. **c** KEGG gene-set enrichment analysis. **d-h** Heat maps of all genes according to KEGG belonging to **d** glutathione metabolism, **e** fatty acid degradation, **f** glucose transport, **g** glycolysis and gluconeogenesis, and **h** ketone body metabolism. All samples derived from RNA from liver tissue of 9 *Mmut*-ki/wt and 10 *Mmut*-ko/ki male mice in the *ad libitu*m fed state.

| **Genes** | **Primer sequences** |
| --- | --- |
| *Acaa1b* | F: 5‘-AGA CAT CTC CGT GGG CAA TG-3‘  R: 5‘-CTG CAG TCC CGA TGA ACA CT-3‘ |
| *Acadl* | F: 5‘-GGT GTT CAT CAC TAA TGG CTG G-3‘  R: 5‘-AGT TCT GCT GTG TCC TGA GC-3‘ |
| *Acadm* | F: 5‘-TGA CAA AAG CGG GGA GTA CC-3‘  R: 5‘-TTT CCG GAA TGT GCG CGT TG-3‘ |
| *Actb* | F: 5‘-GGT GGG AAT GGG TCA GAA GG-3‘  R: 5‘-AGG TCT CAA ACA TGA TCT GGG T-3‘ |
| *Cyp4a10* | F: 5‘-GAC CTA CCT CCA GGC CAT TG-3‘  R: 5‘-AGC TTG ATC ACT CCA TCT GTG T-3‘ |
| *Cyp4a31* | F: 5‘-CCG GAA GAT GCT AAC CCC AG-3‘  R: 5‘-GCC GTT CCC ATT TGT CTA GC-3‘ |
| *Ehhadh* | F: 5‘-ACA ACT TCT GTG CAG GTG CT-3‘  R: 5‘-GAA GCC AAC ACG AGC CTT TG-3‘ |
| *Fgf21* | F: 5‘-GCT CTC TAT GGA TCG CCT CAC-3‘  R: 5‘-GAG TCA GGA CGC ATA GCT GG-3‘ |
| *Hmgcl* | F: 5‘-AGG CTT TGA GGA AGC GGT AG-3‘  R: 5‘-CTT TAG CCG GGG AGA CCT TC-3‘ |
| *Hmgcs1* | F: 5‘-TCT ACC GCA AAA AGA TCC GTG-3‘  R: 5‘-TCT AAT TTA ACG TCC CCA AAG GC-3‘ |
| *Hnf4a* | F: 5‘-CTG TCC CAG CAG ATC ACC TC-3‘  R: 5‘-TCA TTG CCT AGG AGC AGC AC-3‘ |
| *Pck1* | F: 5‘-TGC ATG AAA GGC CGC ACC-3‘  R: 5‘-GTT GCA GGC CCA GTT GTT G-3‘ |
| *Pklr* | F: 5‘-GGG TGA CCT TGG CAT TGA GA-3‘  R: 5‘-TGC TCT CCA GCA TCT GTG TG-3‘ |
| *Ppargc1a* | F: 5‘-CCT CAC ACC AAA CCC ACA GA-3‘  R: 5‘-GAG GAG TTA GGC CTG CAG TT-3‘ |

**Supplementary Table 1: List of primer sequences used for qRT-PCR analysis.** F: forward primer. R: reverse primer
